# BOG: R-package for Bacterium and virus analysis of Orthologous Groups

**DOI:** 10.1016/j.csbj.2015.05.002

**Published:** 2015-05-21

**Authors:** Jincheol Park, Cenny Taslim, Shili Lin

**Affiliations:** a Department of Statistics, Keimyung University, South Korea; b Ohio State University Medical Center, USA; c Department of Statistics, State University, USA

**Keywords:** Bacterium and virus analysis, Clusters of Orthologous Groups, Hypergeometric test, Mann–Whitney Rank Sum test, Gene set enrichment analysis, Tabular and graphical visualization

## Abstract

BOG (Bacterium and virus analysis of Orthologous Groups) is a package for identifying groups of differentially regulated genes in the light of gene functions for various virus and bacteria genomes. It is designed to identify Clusters of Orthologous Groups (COGs) that are enriched among genes that have gone through significant changes under different conditions. This would contribute to the detection of pathogens, an important scientific research area of relevance in uncovering bioterrorism, among others. Particular statistical analyses include hypergeometric, Mann–Whitney rank sum, and gene set enrichment. Results from the analyses are organized and presented in tabular and graphical forms for ease of understanding and dissemination of results. BOG is implemented as an R-package, which is available from CRAN or can be downloaded from http://www.stat.osu.edu/~statgen/SOFTWARE/BOG/.

## 1 Introduction

BOG (Bacterium and virus analysis of Orthologous Groups) is an R-package for identifying groups of differentially regulated genes in the light of gene functions for various virus and bacteria genomes. BOG can be useful in transcriptional profiling of virulent pathogens taking into account of functional categories, an important scientific research area of relevance to detection of bioterrorism. For example, in human host, the concentration of free iron available to bacterium controls the pathogen growth. Effective strategies for adaptation to this altered environmental conditions and, subsequently, the acquisition of iron, are vital to the survival of most bacterial pathogens. Many pathogens undergo significant changes in their gene and protein expression to adapt to growth in iron limiting conditions, including *Bacillus anthracis*, the causative agent of anthrax, a highly virulent pathogen that has been used in recent history as a biological weapon [3]. BOG may also be applicable to studies of marine ecosystems. An example is the study of how hydrostatic pressure may impact the transcriptome of a deep-sea indigenous organism, *Desulfovibrio hydrothermalis*[1]. Such a study is critical in understanding the marine ecosystems, especially those of the deep sea, which represent a major volume of the biosphere. Other examples include bacterial biofilms, important for the study of resistance to antibiotics [6], and *Brassica napus*, an important oil crop [4].

For the type of studies discussed above, the typical first step is to profile the entire transcriptome to identify genes that are differentially expressed (DE) under different conditions (e.g. iron depleted vs. iron replenished in *B*. *anthracis*, or in situ hydrostatic pressure vs. atmospheric pressure in *D*. *hydrothermalis*). For this task, many software packages are available, including DEseq [2], EdgeR [8], Cufflinks [14], and DIME [5]. However, finding the set of DE genes is typically not the end goal. Rather, the interest is to find Clusters of Orthologous Groups (COGs) that are enriched (i.e. over-represented) among the DE genes identified in the first step. This, the second, step is essential for providing new insights into the underlying molecular mechanisms linked to the adaptation of a bacterium or a virus from a native to a perturbed condition. Despite the critical importance of this task, studies of this nature are largely descriptive rather than inferential. Pie charts and bar graphs are often the only tools used to visually depict COGs having a larger share of the DE genes, which are then interpreted as indication of enrichment [3,1,4,6]. However, this does not take into account the sizes of COGs, which can be problematic as a larger share of the DE genes may not be that unusual if the corresponding COG also contains more genes. Further, the descriptive nature of the methods does not lead to conclusions that are based on proper evaluation of scientific evidence. Despite an abundance of software for finding DE genes, to the best of our knowledge, there is no computational tool/software currently available for identifying COGs that are significantly enriched with DE genes. Although such an analysis is similar to finding gene ontology (GO) functional categories that are significantly enriched, a software package for such a purpose, such as GOTM [15], is not directly applicable to finding COGs that are over represented among DE genes. Hence, we believe that it is of value for a software package like BOG that is capable of quick and accurate identification of COGs that are over-represented among differentially expressed genes through rigorous statistical tests.

**Fig. 1:**
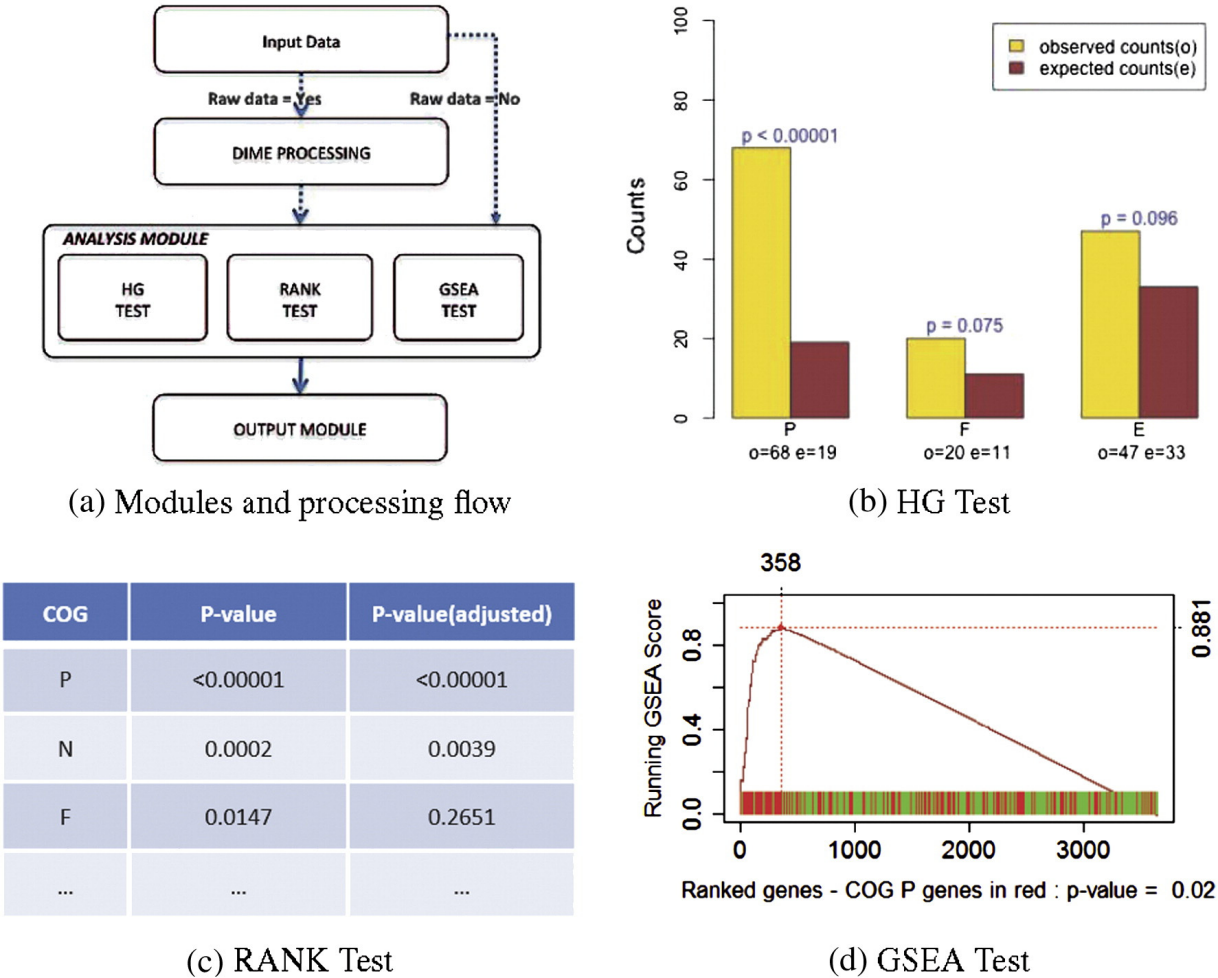
Flowchart and sample outputs. (a) The flowchart depicts the three sequential modules that made up BOG. (b) COGs with adjusted p-value < 0.1 from the hypergeometric test. For each COG (P, F and E), the left bar represents the observed number of differentially expressed genes identified, while the right bar is for the expected number according to the size of the COG. The p-values indicated are adjusted p-value taking into account of multiple testing. (c) Tabular outcome from the Mann–Whitney rank test. The middle column gives raw p-values, while the last column provides adjusted p-values taking multiple testing into consideration. (d) An example GSEA scoring path for the “P” category. One can see the maximum score is reached at 358 genes, with the majority of the genes in the top 358 coming from the “P” category (in red). The p-value is adjusted for multiple testing. (For interpretation of the references to color in this figure legend, the reader is referred to the web version of this article.)

BOG consists of three modules: (optional) DIME processing, analysis, and output modules (Fig. 1(a)). More specifically, after reading in a raw input data set, BOG performs a differential analysis through a mixture ensemble procedure and computes local fdr as a differential score for each gene using the DIME software (http://cran.r-project.org/web/packages/DIME/) [11,12]. If the input data are already (adjusted) p-values rather than raw data, then BOG will skip the DIME preprocessing step. The scores (either calculated or as input) are delivered to the analysis module, which performs three alternative statistical tests to identify COGs that are over represented among the differentially expressed genes: hypergeometric, Mann–Whitney, and gene set enrichment analysis. The analysis results will then be delivered to the output module for tabular and graphical presentation for ease of understanding and dissemination of results.

## 2 Statistical tests in the analysis module

Suppose we have a list of genes $G=\left\{g_{1},\ldots ,g_{N}\right\}$ in an experiment; their associated memberships with a set of known orthologous groups (*M*) are denoted by ℳ = {*m*(*g*_1_), …, *m*(*g*_*N*_) : *m*(*g*_*i*_) ∈ *M*}. We also attach to each gene a differential score *s*(*g*_*i*_) (local fdr or p-values): $S=\left\{s\left(g_{1}\right),\ldots ,s\left(g_{n}\right)\right\}$, which are either obtained directly from user's input or computed by DIME. For each orthologous group *m* ∈ *M* with the corresponding gene set $G_{m}=\left\{g_{i}:m\left(g_{i}\right)=m\right\}$, we denote its size by $n_{m}=||G_{m}||$. In the following, we describe each of the three analysis methods.

### 2.1 Hypegeometric (HG)

We let *K* be the number of genes that are deemed to be differentially expressed under two conditions, that is, *K* = ∑_*i*_^*N*^*I*{*s*(*g*_*i*_) < s*}, where s* is a preset threshold (default is set to be 0.05 on BOG but can be changed by user) and *I*{⋅} is the usual indicator function taking the value of 1 or 0. For each orthologous group *m* ∈ *M*, under the null hypothesis that this group is not over-represented among the set of differentially expressed genes, the test statistic $T_{HG}=\sum_{g_{i}\in G_{m}}I\left\{s\left(g_{i}\right)<s^{*}\right\}$ follows the HG distribution *H*(*K*, *N*, *n*_*m*_). The null hypothesis is rejected if the associated p-value is small, that is, *T* is much larger than what one would expect under the HG distribution.

### 2.2 Mann–Whitney Rank Sum (RANK)

To avoid the need to preset a “significance” threshold (which is somewhat arbitrary), we consider all genes by using their rankings based on their differential scores. Specifically, for each gene $g_{i}\in G,i=1,\ldots ,N$, we assign it a ranking *r*(*g*_*i*_)≡*r*{*s*(*g*_*i*_)} such that a gene with a smaller score will be assigned a higher rank (large number). For each orthologous group *m* ∈ *M*, we compute the test statistic $T_{\mathrm{RANK}}=\sum_{g_{i}\in G_{m}}r\left(g_{i}\right)$. Under the null hypothesis that this group is not over-represented among the set of differentially expressed genes, the expected value of *T*_*RANK*_ is *n*_*m*_(*N* + 1)/2. If the observed statistic is significantly larger than this expected value, then this orthologous group is deemed over-represented.

### 2.3 Gene set enrichment analysis (GSEA)

Instead of basing on correlations as in the original GSEA [9], the modified GSEA in this paper uses rankings of the scores for all genes, like the RANK test. As such, there is no need to preset a threshold of significance. However, unlike the RANK test, evidence of over representation of a COG is evaluated in a sequential manner. More specifically, let $\tilde{G}=\left\{{\tilde{g}}_{1},\ldots ,{\tilde{g}}_{N}:{\tilde{g}}_{i}\in G\right\}$ be the ordered set of genes such that $r\left({\tilde{g}}_{1}\right)\geq \ldots \geq r\left({\tilde{g}}_{N}\right)$. Recall that a smaller score will receive a higher ranking value. For each orthologous group *m* ∈ *M*, we evaluate the sequences of the *P*_*i*_^+^ and *P*_*i*_^−^ values, *i* = 1, ⋯ *N*:

(1) $$P_{i}^{+}\left(G_{m}\right)=\sum_{k\in K_{m}\left(i\right)}\frac{r\left({\tilde{g}}_{k}\right)}{r_{m}},P_{i}^{-}\left(G_{m}\right)=\sum_{k\notin K_{m}\left(i\right),k\leq i}\frac{1}{N-n_{m}}\text{,}$$

where $K_{m}\left(i\right)=\left\{k\leq i:{\tilde{g}}_{k}\in G_{m}\right\}$, $r_{m}=\sum {{}_{\tilde{g}k\in G}}_{m}r\left(\tilde{g}k\right)$. We define the GSEA statistic as $T_{\mathrm{GSEA}}=\max_{i}\left\{P_{i}^{+}\left(G_{m}\right)-P_{i}^{-}\left(G_{m}\right)\right\}$. Its associated p-value for evaluating evidence of over representation of differential expression of genes in *m* is determined by a permutation test by randomly permuting the *N* gene labels.

## 3 BOG software package

We briefly describe the main functions and data input. More details, especially on control parameters of functions, are available in the documentation of the BOG package. The package can be downloaded from CRAN or from http://www.stat.osu.edu/ statgen/SOFTWARE/BOG/. BOG is the flagship function that performs the HG, RANK, and GSEA tests. It takes two primary arguments:

• data: BOG accepts a data file (R dataframe) of two columns. The first column is the geneIDs (characters) and the second is numerical measures for the corresponding genes, which has two possible options controlled by the data. type argument: (1) “data”, normalized “differences” of gene expressions between two comparison groups, (2) “pval”, (adjusted) p-values for each gene if differential analysis is carried out beforehand. Under option (1), BOG assumes that the data are already normalized. Further, “difference” is in a broad sense, which can either be log-difference or just difference without performing log-transformation first, depending on the preference of the user and the context of the problem [10].
• cog_file: This can either be a user specified input file (R dataframe) or simply the specification of the name of one of the built-in COGs: anthracis, brucella, coxiella, difficile, ecoli, or francisella [13]. More specifically, if the virus/bacterium being analyzed is not one of the six build-in varieties, then a file with two columns is required: the first column provides gene IDs as in the input data file; the second column specifies the Clusters of Orthologous Groups to which each gene belongs.

The output module receives results from the analysis module and summarizes them in a tabular format with three columns: COG, p-value, and adjusted p-value, for each of the tests performed. A user can display the table by running the command printHG, printRANK, or printGSEA. Further, BOG provides several graphical functions for visualizing the results, including hgplot and gseaplot.

## 4 Example

To demonstrate the use of BOG, we analyze a set of gene expression levels of *B*. *anthracis* grown in iron depleted media (0μM iron concentration) and iron replenished media (30μM iron concentration) at the four hour time point after treatment [3].

To identify genes whose expressions are altered when iron is depleted, we took the average difference of normalized gene expression values at 0 μM vs. 30 μM after 4 h of treatment (each with four replicates). We first ran DIME to analyze the data and obtain the local fdr value for each of the genes. This list of fdr value was then saved as input to BOG and made available in the BOG package as input file *anthracis*_*iron*. We chose to demonstrate our example in a “piecemeal” fashion to facilitate greater understanding. We ran the following command with the BOG main function to analyze over representation:

$$\begin{array}{l} \mathrm{bog}<‐\mathrm{BOG}(\mathrm{data}=“\mathrm{anthracis}\_\mathrm{iron}”,\;\mathrm{data}.\mathrm{type}=\;“\mathrm{pval}”,\\ \mathrm{cog}.\mathrm{file}=\;“\mathrm{anthracis}”,\;\mathrm{hg}.\mathrm{thresh}\;=\;0.01,\;\mathrm{gsea}=\;\mathrm{TRUE})\text{.} \end{array}$$

The output in *bog* is then processed using various function in the Output model and the results are presented in Fig. 1(b–d). Output from the HG test, summarized using hgplot(bog), is visualized in Fig. 1(b) for COGs with (adjusted) p-value < 0.1. From the results, we can see that “P” (inorganic ion transport and metabolism) is the most significant COG. The results from the RANK test are being summarized in a tabular form (Fig. 1(c)) using the command printRANK(bog), which shows that COG “P” is also returned as the most significant. Finally, we demonstrate the GSEA-path for category “P” in Fig. 1(d) by using the command gseaplot(bog, “ P ”), from which one can see that this category is being selected as over-represented among genes that are differentially expressed in iron depleted condition against iron repleted one. The consistent results from all three tests are reassuring. More importantly, this finding is also consistent with current understanding of the science, as the significant increase in ion transport mechanism and some aspects of matabolism is a clear indication of adaptation to growth under iron depleted condition [3].

## 5 Discussion

We develop an R package (BOG) for identification of Clusters of Orthologous Groups in bacteria and viruses that are enriched among genes that have gone through significant changes under different conditions. Three tests are available to provide user with greater choices. Hypergeometric and Mann–Whitney rank tests are computationally efficient, although note that the hypergeometic test requires the specification of a “significance” threshold. On the other hand, the gene set enrichment analysis based on fdr instead of correlation as in [9] does not need the specification of a threshold, but it is computationally intensive. Therefore, the package provides user with the flexibility of whether to run the GSEA option. As we demonstrated through application to the *B*. *anthracis* example, all three tests consistently identified the same category as the most enriched gene set. For convenience, we use gene expression as our example data type, although BOG is also applicable to other high-throughput data, including DNA-protein binding and methylation data. For the initial step of finding DE genes, we use DIME as the default in BOG, although this can be replaced by any other package including those mentioned in Section 1. The software is written in such a way that the step for finding DE genes can be performed using a user-desired software before calling BOG to identify COGs that are enriched among the set of DE genes. As such, BOG is directly applicable to all examples discussed in Section 1; the set of DE genes or the rankings can be used as input to BOG to formally test which BOGs are enriched in addition to simple descriptive statistics/graphs used therein. In addition to its intended use in detection of pathogens, BOG might also find applications in analyzing gut microbiota community compositions, a subject with recent surge of interests, as such compositions may be related to obesity and other health conditions [7]. For instance, in an analysis of 16S rRNA gene from a study of obese and lean individuals, one may first detect taxa, at a particular taxonomic rank (e.g. species), that have significantly different proportions among these two groups of individuals. Then BOG can be called to identify categories at a higher taxonomic rank (e.g. family) that are significantly enriched.
